# To the Medical Profession

**Published:** 1873-03

**Authors:** J. F. Alexander

**Affiliations:** Chairman Committe of Arrangements.; Atlanta, Georgia


### GEORGIA MEDICAL ASSOCIATION.

### TO THE MEDICAL PROFESSION.

ATLANTA, GEORGIA, March 21, 1872.

I desire to say to the regular medical profession of Georgia that the Committee of Arrangements, under the direction of the profession here, and by their liberality and that of the citizens of this city, are preparing a reception upon a scale proportioned to the entertainment of not only every member of the Georgia Medical Association, but of every regular medical man in the State. We want a large and full representation from every section, and the inauguration of a new era in the history of the body, which will soon put it upon as high a footing, as a scientific body, as any other medical organization in the land.

J. F. ALEXANDER, M.D.,
*Chairman Committe of Arrangements.*

